# A fungal cell wall elicitor from *Neopestalotiopsis clavispora* induces systemic defense in *Ginkgo Biloba*

**DOI:** 10.1186/s12870-026-08202-9

**Published:** 2026-01-22

**Authors:** Yuqing Dong, Shumin Han, Jiasui Zhan, Tianhui Zhu

**Affiliations:** 1 College of Forestry, Sichuan Agricultural University, Chengdu, Sichuan 611130 China; 2 Department of Forest Mycology and Plant Pathology, Swedish University of Agricultural Sciences, Uppsala, 75007 Sweden; 3 Forest Ecology and Conservation in the Upper Reaches of the Yangtze River Key Laboratory of Sichuan Province, Chengdu, Sichuan 611130 China; 4 Sichuan Mt. Emei Forest Ecosystem National Observation and Research Station, Chengdu, Sichuan 611130 China

**Keywords:** Plant immunity, Defense priming, Fungal elicitors, Ginkgo biloba, Pathogen-associated molecular patterns (PAMPs)

## Abstract

**Background:**

Sustainable management of tree diseases requires harnessing the plant’s own immune system. Leaf blight caused by *Neopestalotiopsis clavispora* (*N. clavispora*) affects the ornamental value of *Ginkgo biloba* (*G. biloba*) and the accumulation of its medicinal components. Elicitors, as a novel biological control method, hold potential application value in the prevention and management of *G. biloba* leaf blight.

**Results:**

We demonstrate that a cell wall elicitor extracted from the fungal pathogen *N. clavispora* potently induces systemic resistance against leaf blight in the ancient gymnosperm *G. biloba*. The elicitor exhibited no direct antifungal activity, confirming that its protective function is mediated exclusively through plant innate immunity. Pre-treatment with the elicitor resulted in over 80% disease control, outperforming commercial resistance inducers and matching the efficacy of carbendazim fungicide. This resistance was associated with a primed state, characterized by a rapid and sustained burst in key defense enzyme activities (POD, PAL, PPO), accelerated accumulation of lignin and phenolics, and mitigated oxidative damage. Metabolomic analyses revealed that the elicitor triggers a change of the defense landscape. We identified coordinated upregulation of the phenylpropanoid pathway, which was directly mirrored by the accumulation of defensive flavonoids and flavonols. Furthermore, tryptophan metabolism and glycerophospholipid pathways were significantly altered, indicating a comprehensive reconfiguration of primary and secondary metabolism.

**Conclusions:**

Our findings uncover a multifaceted defense strategy in *G. biloba*, wherein the fungal elicitor acts as a priming agent to establish a state of alert, enabling a robust, integrated metabolic response that effectively restricts pathogen invasion. This work provides a molecular framework for elicitor-induced resistance in trees and validates a sustainable, vaccine-like strategy for forest protection.

## Background

*Ginkgo biloba L.*, a renowned living fossil, represents a unique evolutionary lineage that has persisted for over 200 million years. As one of the oldest extant tree species, it offers an invaluable window into the evolution of plant defense mechanisms [1]. Beyond its profound phylogenetic significance, *G. biloba* is a species of considerable economic and ecological importance, cultivated worldwide for its ornamental value and medicinal metabolites such as flavonoids and terpene lactones [2]. However, the health and productivity of this ancient species are increasingly threatened by fungal diseases including leaf spot and leaf blight [3, 4].

Among these, leaf blight caused by the ascomycete *Neopestalotiopsis clavispora* is particularly destructive leading to severe defoliation, reduced tree vitality and significant economic losses [5]. This pathogen demonstrates a broad host range. It can infect numerous economically important plants globally [6, 7], underscoring its adaptive potential and the urgent need for effective, sustainable control strategies.

Conventional management of fungal diseases in forestry and horticulture has heavily relied on chemical fungicides. While often effective, their continuous use poses environmental risks, can lead to the selection of resistant pathogen strains and is increasingly at odds with global demands for sustainable agriculture and silviculture [8]. Consequently, there is a pressing need to develop alternative strategies that harness the plant’s own innate immune system. A particularly promising approach involves the use of elicitors to prime the defensive capacity of plants, offering a prophylactic, eco-friendly alternative to traditional chemicals [9, 10].

The plant immune system is a sophisticated two-tiered detection network. The first layer, Pattern-Triggered Immunity (PTI), is activated upon recognition of conserved microbial signatures, known as Pathogen-Associated Molecular Patterns (PAMPs), by surface-localized Pattern Recognition Receptors (PRRs) [11]. Fungal cell walls are a rich source of such PAMPs, including chitin, β-glucans, and glycoproteins [12, 13]. Their recognition initiates a robust defense signaling cascade involving reactive oxygen species (ROS) bursts, mitogen-activated protein kinase (MAPK) activation, stomatal closure, and extensive transcriptional reprogramming that reinforces cell walls and amplifies the production of antimicrobial compounds [14–16]. A key feature of this induced state is systemic acquired resistance (SAR), which provides long-lasting, broad-spectrum protection against subsequent pathogen challenges.

Critically, exposure to specific elicitors can establish a physiological state known as “defense priming.” In this primed state, plants do not constitutively activate costly defense responses but are poised to mount a faster and stronger defense upon pathogen perception, thereby enhancing resistance without major fitness costs [10]. This phenomenon, analogous to vaccination in animals, is mediated by epigenetic and metabolic changes that create a “memory” of the initial stimulus. The application of fungal elicitors has been successfully demonstrated to prime defense responses in various herbaceous crops, leading to enhanced resistance through the upregulation of defense-related genes and metabolic pathways [17–19]. However, the molecular and metabolic mechanisms underlying this primed state, particularly in long-lived perennial trees, remain far less elucidated.

Trees, with their extended lifespans and sedentary nature, have evolved distinct defense strategies compared to annual plants [20]. *G. biloba*, as a gymnosperm, occupies a pivotal phylogenetic position between ferns and angiosperms, yet the molecular basis of its immune responses is profoundly understudied. A significant knowledge gap exists in our understanding of how tree immune systems, especially in non-model species like *G. biloba*, perceive pathogen-derived signals and translate them into a coordinated transcriptional and metabolic defense output. While the efficacy of exogenous elicitors is established in some systems [15, 21], the specific mechanisms are often opaque. Furthermore, the use of a homologous elicitor (i.e., derived from the pathogen itself) to protect against the same pathogen is an underexplored strategy that may offer high specificity. Therefore, we focused this investigation specifically on the *G. biloba - N. clavispora* pathosystem. This targeted approach allows us to address a concrete phytopathological challenge while exploring the immune mechanics of a phylogenetically unique gymnosperm, whose defense signaling may diverge from that of well-characterized angiosperm models.

In this study, we sought to bridge this gap by investigating the capacity of a cell wall elicitor from *N. clavispora* to induce resistance against leaf blight in *G. biloba.* We hypothesized that this fungal elicitor would act as a PAMP, priming the *G. biloba* immune system for a more effective defense without direct antimicrobial activity. To test this, we first confirmed the lack of direct fungicidal effects and then quantified the elicitor’s efficacy against pathogen challenge, comparing it to commercial chemical and biological agents. We further conducted a time-resolved physiological analysis to dissect the activation of key defense enzymes and the accumulation of defensive compounds. Finally, to uncover the fundamental mechanistic basis of this induced resistance, we employed an untargeted metabolomics approach to globally profile the mechanisms associated with the elicitor-primed state. Our integrated findings provide a systems-level view of induced resistance in an ancient tree species, revealing that the fungal elicitor orchestrates a robust defense phenotype through the synergistic priming of antioxidant systems and the extensive changes of primary and secondary metabolism, with a marked emphasis on the flavonoid and phenylpropanoid pathways. This work not only advances our understanding of plant immunity in trees but also establishes a sustainable, priming-based strategy for the protection of a cherished living fossil.

## Methods

### Material

*N. clavispora* was isolated from diseased leaf tissue exhibiting *G. biloba* leaf blight (*ITS* accession: OQ152504.1; *TUB2* accession: OQ168328.1; *TEF1-α* accession: OQ168329.1). Two-year-old *G. biloba*, procured from Shuyang County, Jiangsu Province, were cultivated in the greenhouse of the College of Forestry, Sichuan Agricultural University. The greenhouse conditions were maintained at 25 ± 3 °C and 70–80% relative humidity. Plant Superoxide Dismutase (SOD) Assay Kit (AKAO001M), Plant Peroxidase (POD) Assay Kit (AKAO005M), Plant Lignin Content Assay Kit (AKSU010M), Plant Total Phenolic Content Assay Kit (AKPL016M), and Malondialdehyde (MDA) Content Assay Kit (AKFA013M) were sourced from Beijing Boxbio Science & Technology Co., Ltd. 

### Preparation of the cell wall elicitors from *N. clavispora*

The cell wall elicitor was prepared from *N. clavispora* mycelia according to established protocol [22, 23] with modifications. Briefly, the fungus was shake-cultured in Potato Dextrose Broth (PDB) for 5 days at 25 °C. The resulting mycelial biomass was harvested by vacuum filtration and subjected to a series of purification steps. The mycelia were thoroughly washed with 50 mmol/L phosphate-buffered saline (PBS, pH 7.0) and subsequently homogenized in PBS containing 0.5% (v/v) Triton X-100. The homogenate was centrifuged, and the pellet was washed with 95% ethanol to remove residual lipids and intracellular contaminants. The purified mycelia were then disrupted using an ultrasonic disruptor on an ice bath. The lysate was centrifuged at 12,000 rpm for 20 min at 4 °C to collect the insoluble cell wall fraction.

The crude cell wall precipitate was mixed with deionized water at a 1:10 (w/v) ratio and hydrolyzed by autoclaving at 121 °C for 2 h to solubilize elicitor-active components. After cooling to room temperature, the mixture was centrifuged again at 12,000 × g for 20 min at 4 °C. The supernatant was collected and sterilized by passage through a 0.45 μm membrane filter. The final filtrate was designated as the *N. clavispora* elicitor stock solution and stored at 4 °C until use.

### Preparation of pathogen spore suspension

A spore suspension of *N. clavispora* was prepared for inoculation assays. The fungus was cultured on Potato Dextrose Agar (PDA) medium and incubated for 20 days at 25 °C to induce sporulation. To harvest spores, the culture surface was gently rinsed with a sterile aqueous solution of 0.05% (v/v) Tween-80, and any adherent spores were subsequently dislodged using a sterilized inoculation shovel. The resulting spore suspension was transferred to a sterile tube and agitated on an orbital shaker at 160 rpm for 30 min at 25 °C to ensure complete spore dispersion and break up spore clusters. The suspension was then filtered through sterile, degreased gauze to remove mycelial fragments and agar debris. The filtrate was collected, and the spore concentration was determined using a hemocytometer. The final concentration was adjusted to 1 × 10⁶ spores/mL with sterile 0.05% Tween-80 solution.

### Spore germination assay

The direct effect of the elicitor on fungal spore germination was evaluated using the concave slide germination method [24]. The spore suspension was prepared in a 0.5% glucose solution to provide a nutrient base for germination. This suspension was mixed at a 1:1 (v/v) ratio with the elicitor solution, which had been serially diluted with sterile water to create treatment solutions with final elicitor concentrations of 25, 50, 100, and 200 µg/mL. A control treatment was prepared by mixing the spore suspension with an equal volume of sterile water.

Aliquots of each mixture were transferred to concave slides, which were then placed in a humid chamber and incubated at 25 °C. Spore germination was assessed at 4, 6, and 8 h post-incubation under a light microscope. A spore was considered germinated when the length of the germ tube exceeded the spore’s diameter. The germination rate was calculated for each sample. The experiment was conducted with three independent biological replicates, with each replicate comprising five technical replicate slides per treatment.

### Mycelial growth assay

The direct effect of the elicitor on the vegetative growth of *N. clavispora* was evaluated using a mycelial growth inhibition assay. The elicitor stock solution (5 mg/mL) was serially diluted and aseptically mixed with molten PDA medium to yield final concentrations of 25, 50, 100, and 200 µg/mL. For the control treatment, an equivalent volume of sterile water was mixed with PDA.

Each plate was centrally inoculated with a 5-mm mycelial plug taken from the actively growing margin of a 7-day-old *N. clavispora* culture. The inoculated plates were incubated at 25 °C, and the radial mycelial growth was monitored. The colony diameter was measured on days 1, 3, and 5 post-inoculation using the cross method, whereby the average of two perpendicular diameters was calculated for each colony [25]. The experiment included five biological replicates (independent cultures) per treatment, with each replicate consisting of five technical replicate plates (*n* = 5). 

### Elicitor-induced resistance assay against *G. biloba* leaf blight

The capacity of the elicitor to induce systemic resistance in *G. biloba* was evaluated under controlled conditions. The elicitor was dissolved in an aqueous solution of 0.05% (v/v) Tween-80 (as a surfactant) to prepare working concentrations of 25, 50, 100, and 200 µg/mL. Two-year-old *G. biloba* seedlings with uniform growth were selected, and the adaxial and abaxial surfaces of the leaves were thoroughly sprayed with the respective elicitor solutions until runoff. The control group (CK) and negative control group (NC) were sprayed with an equivalent volume of sterile water containing 0.05% Tween-80. This resulted in a total of six distinct treatment groups.

The experimental design was a randomized complete block with three independent biological replicates per treatment. Each biological replicate consisted of three individual seedlings (for a total of nine seedlings per treatment group), and ten uniform, undamaged leaves per seedling were marked for subsequent inoculation and assessment.

Pathogen challenge was performed 72 h after the elicitor application to allow for the establishment of induced resistance. Inoculation was carried out using a *N. clavispora* spore suspension (1 × 10⁶ spores/mL), the NC group was inoculated with an equivalent volume of sterile water. To facilitate consistent infection, the leaf margins were gently wounded by lightly abrading with sterilized sandpaper, simulating natural entry points as previously described [5]. Disease progression was monitored, and the disease severity was assessed 14 days post-inoculation. The disease index and the relative control efficacy for each treatment were calculated based on standardized visual assessment of lesion development.

### Comparative efficacy of elicitor against commercial agents

The protective efficacy of the fungal elicitor was benchmarked against a panel of commercially available chemical fungicides and resistance inducers. The four chemical agents used for comparison were as follows: 50% carbendazim (applied at 500 µg/mL), 80% mancozeb (800 µg/mL), 43% tebuconazole (300 µg/mL), and 70% thiophanate-methyl (800 µg/mL). Four resistance inducers were 6% oligosaccharin (125 µg/mL), 0.5% chitosan (100 µg/mL), a commercial plant hypersensitive protein (75 µg/mL), and chitosan oligosaccharide (75 µg/mL). All treatment solutions, including the *N. clavispora* elicitor, were prepared in an aqueous solution of 0.05% (v/v) Tween-80 to ensure uniform wetting and foliar absorption. Uniformly grown *G. biloba* seedlings were selected and randomly assigned to treatment groups. The adaxial and abaxial surfaces of all leaves were sprayed to runoff with their respective solutions. A control group sprayed with the 0.05% Tween-80 solution alone was included.

To establish a robust induced state, agents were applied three times at 24-hour intervals. Immediately following each spray, treated plants were enclosed in transparent plastic bags for 24 h to maintain high humidity and promote foliar absorption and systemic translocation of the agents. Pathogen inoculation with *N. clavispora* (1 × 10⁶ spores/mL) was performed 72 h after the final application, using the standardized wounding method described previously. Disease severity was assessed 14 days post-inoculation, and the disease index and relative control efficacy were calculated for each treatment. The experiment employed a randomized block design with three independent biological replicates per treatment.

### Analysis of defense-related physiological biomarkers

To dissect the dynamic physiological responses underlying induced resistance, we quantified the activities of key defense enzymes, oxidative stress markers, and phytoalexins in *G. biloba* leaves following various treatments. The experiment comprised six distinct treatment groups: Sprayed with water, not inoculated with pathogen (CK-1); Sprayed with water, inoculated with pathogen (CK-2); Sprayed with elicitor (100 µg/mL), not inoculated with pathogen (T-1)、Sprayed with elicitor (100 µg/mL), inoculated with pathogen (T-2)、Sprayed with chitosan oligosaccharide (75 µg/mL), inoculated with pathogen (T-3), and Sprayed with 50% carbendazim (500 µg/mL), inoculated with pathogen (T-4). The pathogen was inoculated 72 h after the application of the treatment. Leaf samples of uniform size were collected from each treatment group at 1, 2, 3, 4, 5, 7, 10, and 15 days post-inoculation (dpi). The collected leaves were immediately rinsed with distilled water to remove surface residues, gently blotted dry, rapidly sectioned, and flash-frozen in liquid nitrogen. All samples were stored at -80 °C until analysis.

The activities of defense enzymes including polyphenol oxidase (PPO), superoxide dismutase (SOD), peroxidase (POD), catalase (CAT), and phenylalanine ammonia-lyase (PAL) were measured. Additionally, the contents of malondialdehyde (MDA), lignin, and total phenolics were determined. All assays were performed using a SpectraMax iD3 microplate reader (Molecular Devices, Shanghai, China) with corresponding commercial assay kits (Beijing Boxbio Science & Technology Co., Ltd., Beijing, China), following the manufacturers’ protocols.

### LC-MS/MS-based metabolomic profiling

Untargeted metabolomic profiling was conducted to investigate systemic metabolic changes in *G. biloba leaves* following elicitor treatment and pathogen challenge. Uniform two-year-old seedlings were selected and randomly assigned to two groups: an elicitor treatment group (J), sprayed with the fungal elicitor at 100 µg/mL, and a control group (CK), sprayed with sterile water. Seventy-two hours post-application, all seedlings were challenge-inoculated with *N. clavispora* spore suspension (1 × 10⁶ spores/mL). Leaf samples were collected 7 days after inoculation, with six independent biological replicates per group.

For metabolite extraction, approximately 50 mg of frozen leaf tissue from each sample was precisely weighed, homogenized to a fine powder in liquid nitrogen using a mortar and pestle, and transferred to a 2 mL microcentrifuge tube. A 6 mm stainless-steel grinding bead was added to each tube, followed by 400 µL of ice-cold extraction solvent (methanol: water, 4:1, v/v) containing 0.02 mg/mL L-2-chlorophenylalanine as an internal standard. The samples were then subjected to mechanical homogenization using a frozen tissue grinder (JXFSTPRP-24, Shanghai Jingxin Industrial Development Co., Ltd.) for 6 min at 50 Hz while maintained at -10 °C. Subsequent low-temperature ultrasonic extraction was performed for 30 min in an ice-water bath (5 °C, 40 kHz). To precipitate proteins and insoluble residues, the extracts were incubated at -20 °C for 30 min and then centrifuged at 13,000 rpm for 15 min at 4 °C. The resulting supernatant was carefully transferred to glass LC injection vials equipped with micro-inserts for subsequent instrumental analysis.

### Instrumental analysis

Chromatographic separation and mass spectrometric detection were performed using an ACQUITY UPLC I-Class system (Waters, Milford, USA) coupled online to a TripleTOF 6600 + quadrupole-time-of-flight mass spectrometer (Sciex, Framingham, USA). Metabolite profiling was conducted in both electrospray ionization (ESI) positive and negative modes to achieve broad metabolite coverage.

Chromatographic separation was achieved on an ACQUITY UPLC HSS T3 column (100 mm × 2.1 mm i.d., 1.8 μm; Waters) maintained at 45 °C. The mobile phase consisted of solvent A (water: acetonitrile, 95:5, v/v, with 0.1% formic acid) and solvent B (acetonitrile: isopropanol: water, 47.5:47.5:5, v/v/v, with 0.1% formic acid). A linear gradient elution program was employed as follows: 0–2 min, 5% B; 2–15 min, 5-100% B; 15–17 min, 100% B; 17–17.1 min, 100-5% B; followed by a 3.9 min re-equilibration at 5% B. The flow rate was 0.40 mL/min and the injection volume was 2 µL.

Mass spectrometric data were acquired in data-dependent acquisition (DDA) mode. The ion source parameters were set as follows: ion source temperature, 500 °C; ion spray voltage, ± 5500 V; curtain gas, 30 psi; nebulizer gas (GS1) and heater gas (GS2), 50 psi. The mass range was set to m/z 50-1250 for both MS and MS/MS scans.

### Data processing and statistical analysis

Raw data files were converted to mzML format and processed using the Progenesis QI software (v3.0, Waters Corporation) for peak picking, alignment, and deconvolution, generating a data matrix of metabolite features (retention time, m/z, and intensity). This matrix was then subjected to multivariate statistical analysis on the Majorbio Cloud Platform (cloud.majorbio.com).

Unsupervised principal component analysis (PCA) was performed to visualize general clustering and outliers. Supervised orthogonal partial least squares-discriminant analysis (OPLS-DA) was used to maximize the separation between the elicitor-treated (J) and control (CK) groups. The model quality was assessed by the parameters R²Y and Q². The variable importance in projection (VIP) from the OPLS-DA model was used to rank the contribution of each metabolite to group separation [26]. Differential metabolites were identified by applying a threshold of VIP > 1.0 and a false discovery rate (FDR)-corrected *p*-value < 0.05 from a Student’s t-test. Metabolites were annotated by searching against the KEGG and HMDB databases. Functional enrichment analysis of these differential metabolites was performed based on the KEGG pathway database, with pathways having a *p*-value < 0.05 considered significantly enriched.

For all defense-related physiological data (defense enzyme activities, MDA, lignin, and phenolic contents), statistical significance among treatment groups at each time point was determined using a one-way analysis of variance (ANOVA). A two-way ANOVA was performed to assess the differences in spore germination rate and mycelial growth both across time points and among treatments. Post-hoc comparisons were conducted using Duncan’s multiple range test, with a significance threshold of *p* < 0.05. These analyses were performed with SPSS Statistics software (v26.0, IBM Corp., Armonk, NY, USA). Data visualization and additional statistical validation were carried out using GraphPad Prism (v10.1.2, GraphPad Software, Boston, MA, USA).

## Results

### The fungal elicitor lacks direct antifungal activity against *N. clavispora*

**Fig. 1:**
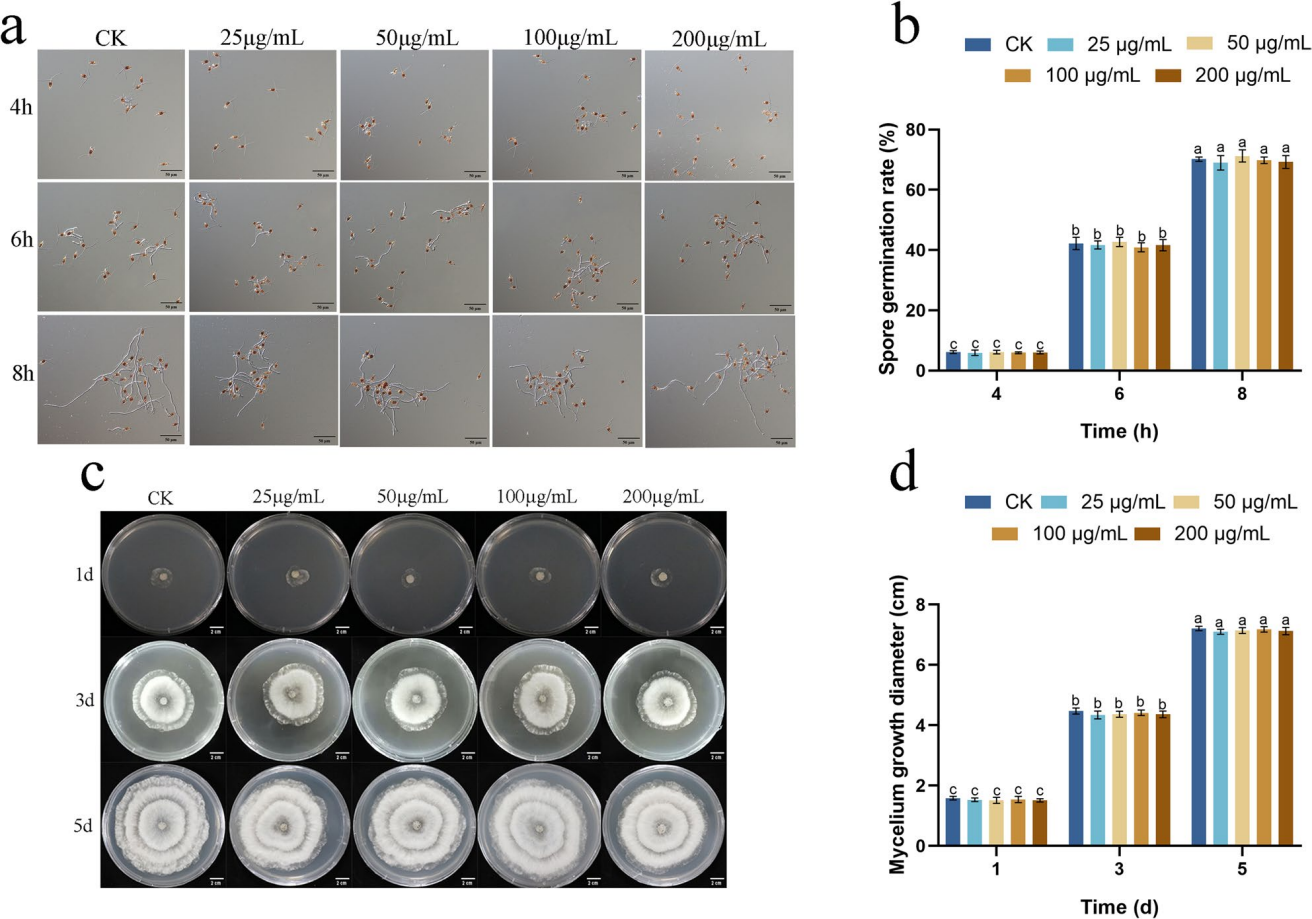
Antifungal activity of the fungal elicitor against *N. clavispora*. **a** germination morphology of *N. clavispora* spores treated with elicitor. **b** Effect of elicitor on spore germination rate. **c** Mycelial growth of *N. clavispora* treated with elicitor. **d** Mycelium diameter of *N. clavispora* treated with elicitor. Different lowercase letters indicate significant differences in different time points and among treatments (*p* < 0.05)

We first assessed whether the cell wall elicitor directly inhibited the growth of *N. clavispora*. Spore germination assays revealed no significant inhibitory effect of the elicitor across a concentration range of 25–200 µg/mL (Fig. 1a, b). After 4 h of incubation, spores across all treatments, including the control, were at initial germination stages with comparable germination rates. Germination progressed rapidly by 6 h, reaching 40.93–42.70% in treated groups, which was not significantly different from the control (42.19%). This trend continued at the 8-hour time point, with germination rates of 68.97–71.26% in treated groups, statistically indistinguishable from the 70.14% observed in the control.

Consistent with the spore germination results, the elicitor also failed to inhibit mycelial growth on PDA medium (Fig. 1c, d). The radial growth of colonies treated with various concentrations of the elicitor showed no significant difference from the control at any measured time point. These results collectively demonstrate that the protective effect of the cell wall extract is not attributable to direct antifungal activity against *N. clavispora* but is instead mediated through the induction of plant resistance. 

### The fungal elicitor potently induces resistance against *G. biloba* leaf blight

**Fig. 2:**
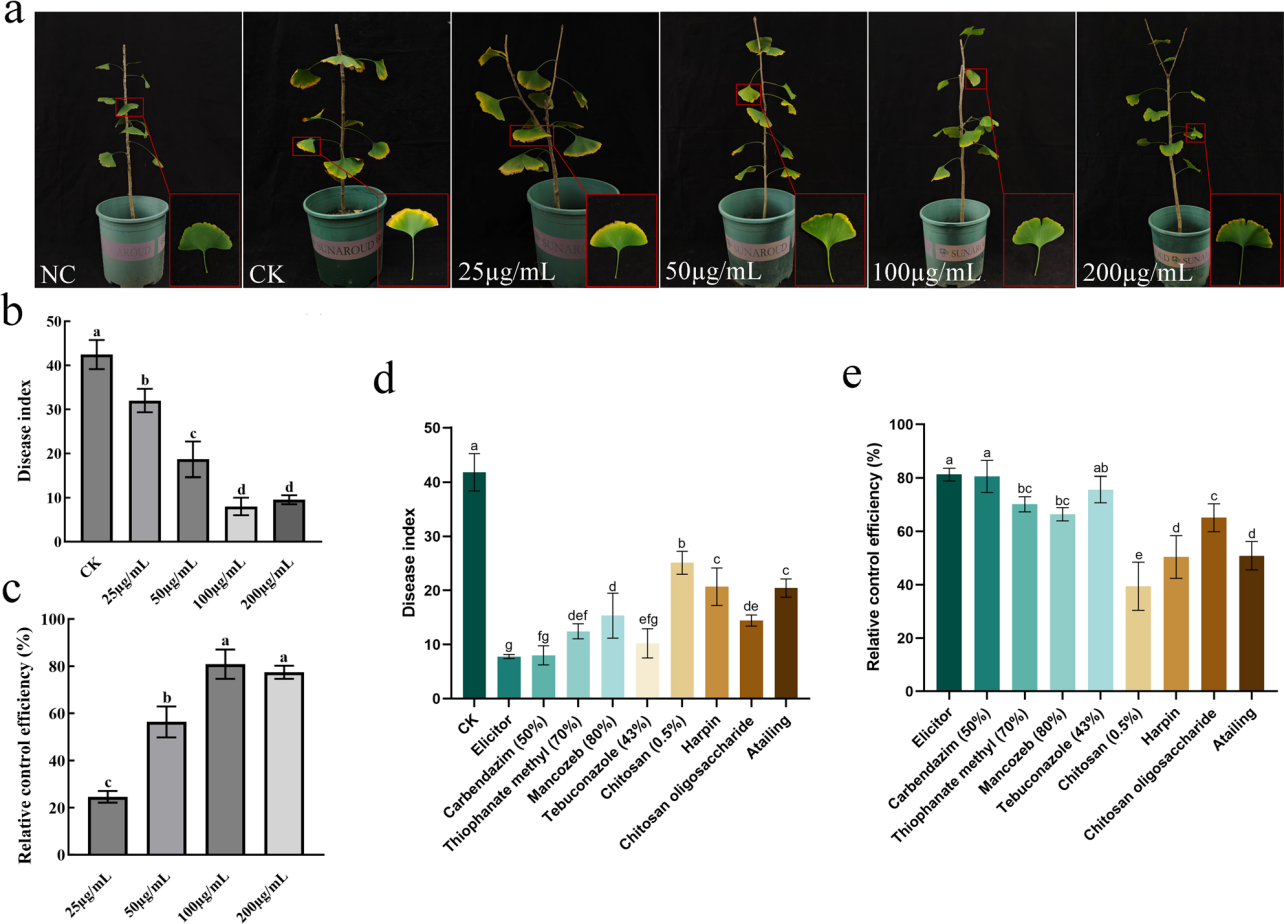
Effect of fungal elicitor treatment on induced resistance against *G. biloba* leaf blight. **a**–**c** Effect of elicitor concentration on disease control: (**a**) Potted plant phenotype, (**b**) disease index, (**c**) relative control efficacy. **d**–**e** Comparison of different treatments: (**d**) disease index, (**e**) relative control efficacy. Different lowercase letters indicate significant differences (*p* < 0.05) among treatments

To evaluate the capacity of the elicitor to induce resistance, *G. biloba* plants were treated with varying concentrations prior to pathogen challenge. The elicitor conferred a strong, concentration-dependent protective effect (Fig. 2a-c). The disease index was negatively correlated with elicitor concentration, with efficacy increasing across the 25–200 µg/mL range before reaching a plateau. Maximum induced resistance was achieved at 100 µg/mL, with a control efficacy of 80.86%. No significant enhancement was observed at 200 µg/mL, indicating a saturation threshold at approximately 100 µg/mL.

We further benchmarked the efficacy of the elicitor against commercial chemical fungicides and resistance inducers. The *N. clavispora* cell wall elicitor demonstrated superior performance, achieving over 80% control efficacy (Fig. 2d, e). This was significantly higher than all tested resistance inducers. Notably, the elicitor’s efficacy was statistically equivalent to the potent fungicide carbendazim (80.55%) and surpassed that of the other three chemical fungicides. These results establish the fungal elicitor as a highly effective resistance inducer, with performance comparable to or better than standard chemical and biological agents. 

### Elicitor treatment primes and enhances defense enzyme activities in *G. biloba*

**Fig. 3:**
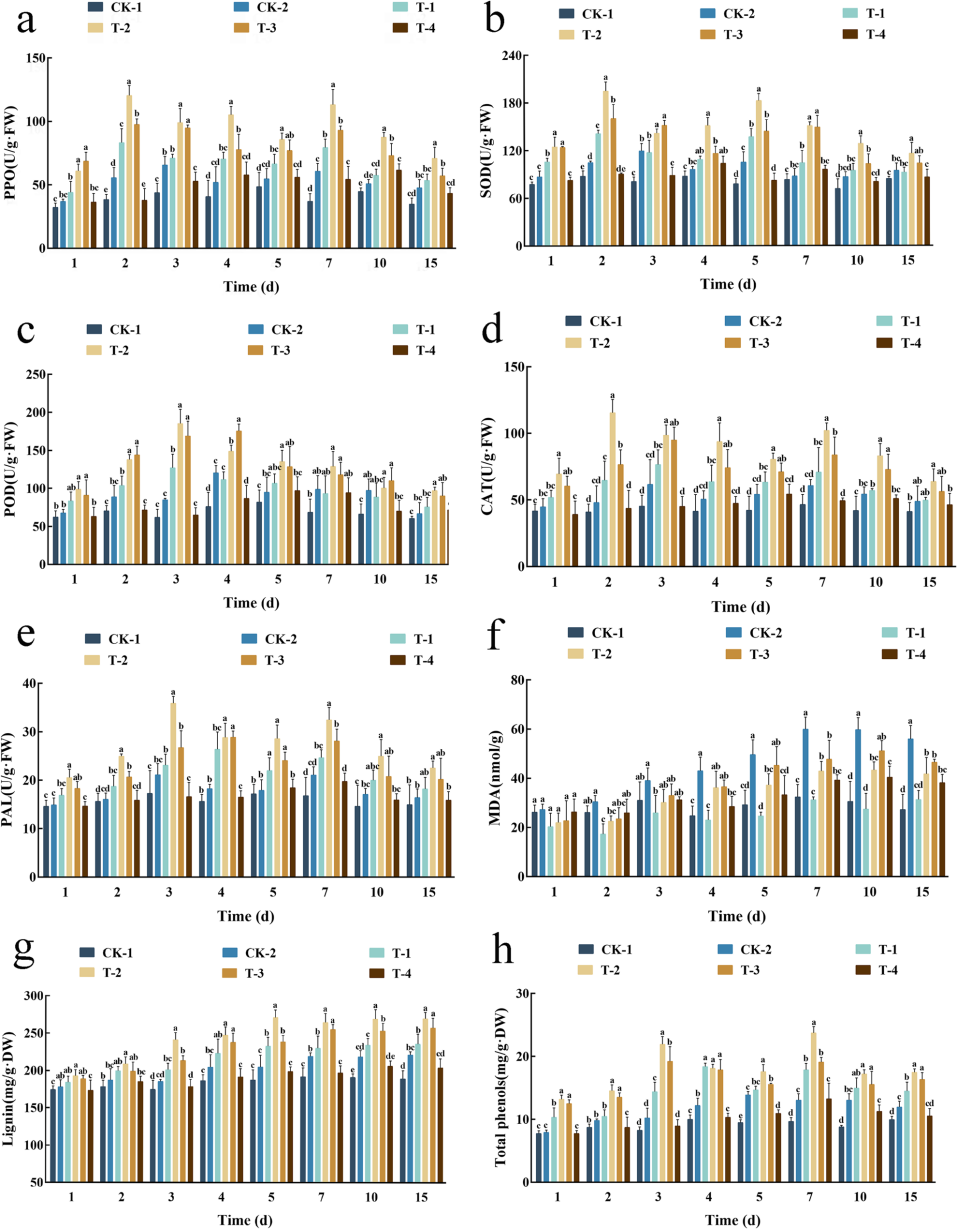
Effect of elicitor treatment on endogenous substance content in G. biloba leaves. **a**–**e** Defense enzyme activities: (**a**) PPO, (**b**) SOD, (**c**) POD, (**d**) CAT, (**e**) PAL. **f** MDA content. **g** Lignin content. **h** Total phenolic content. Different lowercase letters indicate significant differences (*p* < 0.05) among treatments on the same day. Treatments: CK-1 (water, no inoculation), CK-2 (water, inoculated), T-1 (elicitor, no inoculation), T-2 (elicitor, inoculated), T-3 (chitosan oligosaccharide, inoculated), T-4 (carbendazim, inoculated)

The application of the fungal elicitor triggered a robust and sustained activation of key defense enzymes in *G. biloba* leaves. While defense enzyme activities in the healthy control group (CK-1) remained stable, all other treatments induced dynamic changes, with the elicitor-treated, pathogen-challenged group (T-2) showing the most pronounced and persistent response (Fig. 3a-e).

Pathogen inoculation alone (CK-2) activated a defense response, characterized by transient peaks in enzyme activities: PPO and CAT showed bimodal peaks (days 3 & 7), SOD and PAL peaked on day 3, and POD peaked on day 4. In stark contrast, the elicitor-primed plants (T-2) mounted a stronger and more rapid defense. POD activity, for instance, peaked on day 3 at a level 2.18-fold higher than the inoculated control (CK-2) and remained significantly elevated. Similarly, CAT activity exhibited enhanced bimodal peaks (2.40-fold and 1.68-fold higher than CK-2), and PAL activity was significantly upregulated from day 1 onward.

Critically, plants treated with the elicitor alone (T-1), without subsequent pathogen challenge, consistently exhibited elevated defense enzyme activities compared to the healthy control (CK-1). This demonstrates that the elicitor itself is sufficient to pre-activate, or “prime,” the plant’s defense signaling pathways, establishing a state of alert that enables a more efficient and powerful response upon pathogen attack.

### Elicitor treatment mitigates pathogen-induced oxidative membrane damage

Measurement of malondialdehyde (MDA) content, a key marker of lipid peroxidation and oxidative membrane damage, revealed that elicitor pre-treatment significantly alleviated cellular injury caused by *N. clavispora* (Fig. 3f). Pathogen infection alone (CK-2) induced severe oxidative stress, with MDA content rising sharply after day 3 and peaking at day 7, remaining significantly elevated throughout the experiment. This indicates substantial and sustained damage to cellular membranes.

In contrast, elicitor-primed plants (T-2) exhibited a markedly different response. While an initial increase in MDA was observed, it peaked later (day 10) and at a lower level than in CK-2, followed by a significant decline in the later stages (days 10–15). This pattern suggests that the primed defense system effectively contained and repaired the oxidative damage over time. Importantly, plants treated only with the elicitor (T-1) maintained MDA levels as low as the healthy control (CK-1), demonstrating that the elicitor itself does not induce oxidative stress but instead primes the plant to manage it more effectively upon challenge.

### Elicitor induction promotes sustained accumulation of structural and antimicrobial compounds

The elicitor treatment significantly enhanced the biosynthesis of key defensive compounds in *G. biloba* leaves, namely lignin and total phenolics (Fig. 3g, h). While lignin content in the healthy control (CK-1) remained stable, all treated groups showed increased lignin accumulation. Notably, the elicitor-treated groups (T-1 and T-2) and the chitosan oligosaccharide control (T-3) achieved and maintained the highest lignin levels, significantly surpassing other treatments.

A similar but more pronounced trend was observed for total phenolic content. The elicitor-induced and pathogen-challenged group (T-2) consistently exhibited the highest phenolic levels throughout the experiment and maintained this elevated state for a prolonged duration. This sustained accumulation of both lignin, a major structural barrier, and antimicrobial phenolics highlights a key mechanism by which the elicitor fortifies the plant’s physical and chemical defenses. 

### Elicitor induction triggers metabolic changes in *G. biloba*

**Fig. 4:**
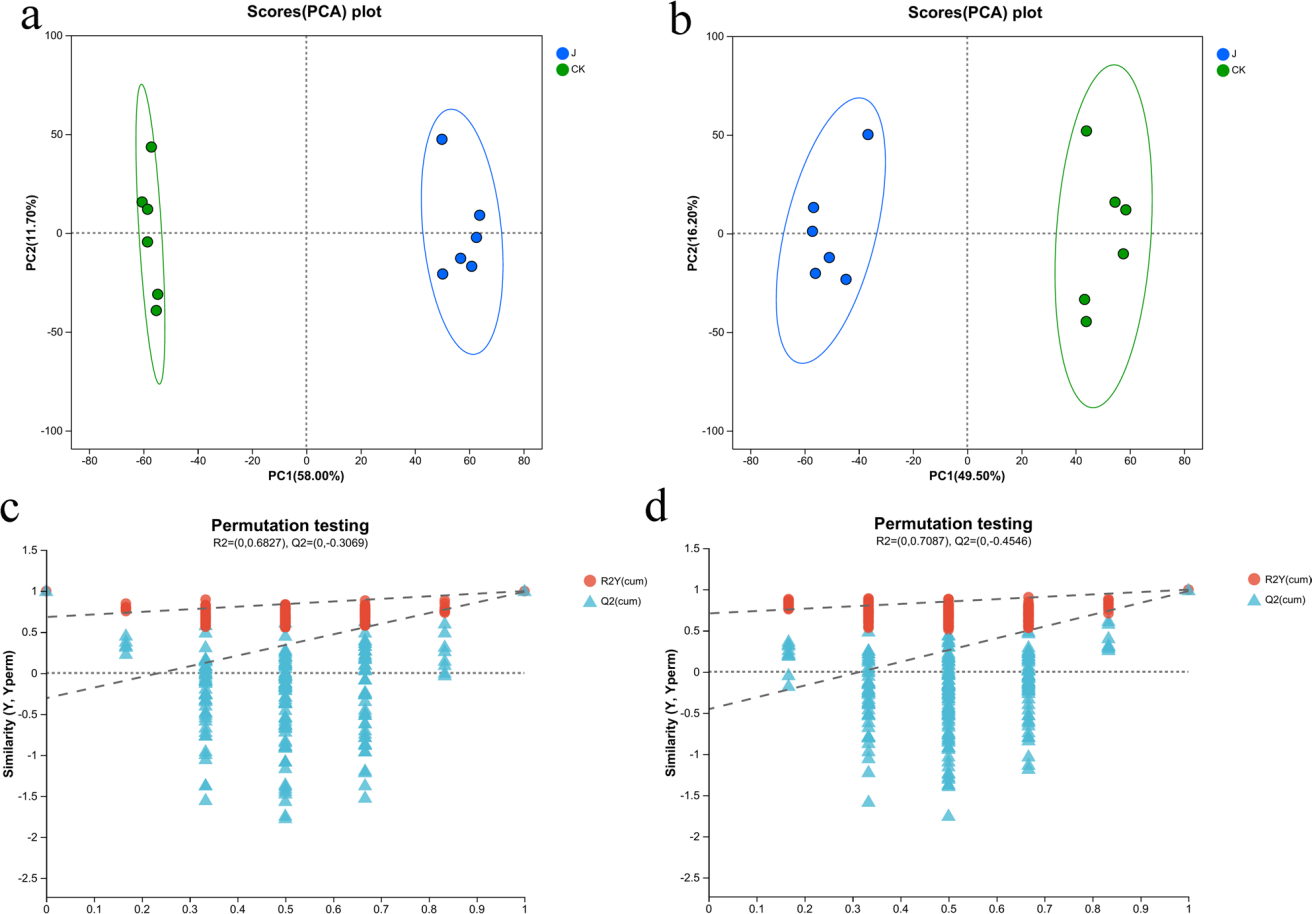
Multivariate statistical analysis of metabolites from *G. biloba* leaves. **a** PCA score plot in positive ion mode; (**b**) PCA score plot in negative ion mode; (**c**) OPLS-DA permutation test in positive ion mode; and (**d**) OPLS-DA permutation test diagram in negative ion mode

To elucidate the metabolic basis of induced resistance, we conducted untargeted LC-MS/MS metabolomic profiling of *G. biloba* leaves. Principal component analysis (PCA) revealed a clear separation between the elicitor-treated (J) and control (CK) groups in both positive and negative ion modes (Fig. 4a, b), indicating a substantial restructuring of the leaf metabolome. This separation was validated by orthogonal partial least squares-discriminant analysis (OPLS-DA), with model validation confirming no overfitting and robust group discrimination (Fig. 4c, d).

**Fig. 5:**
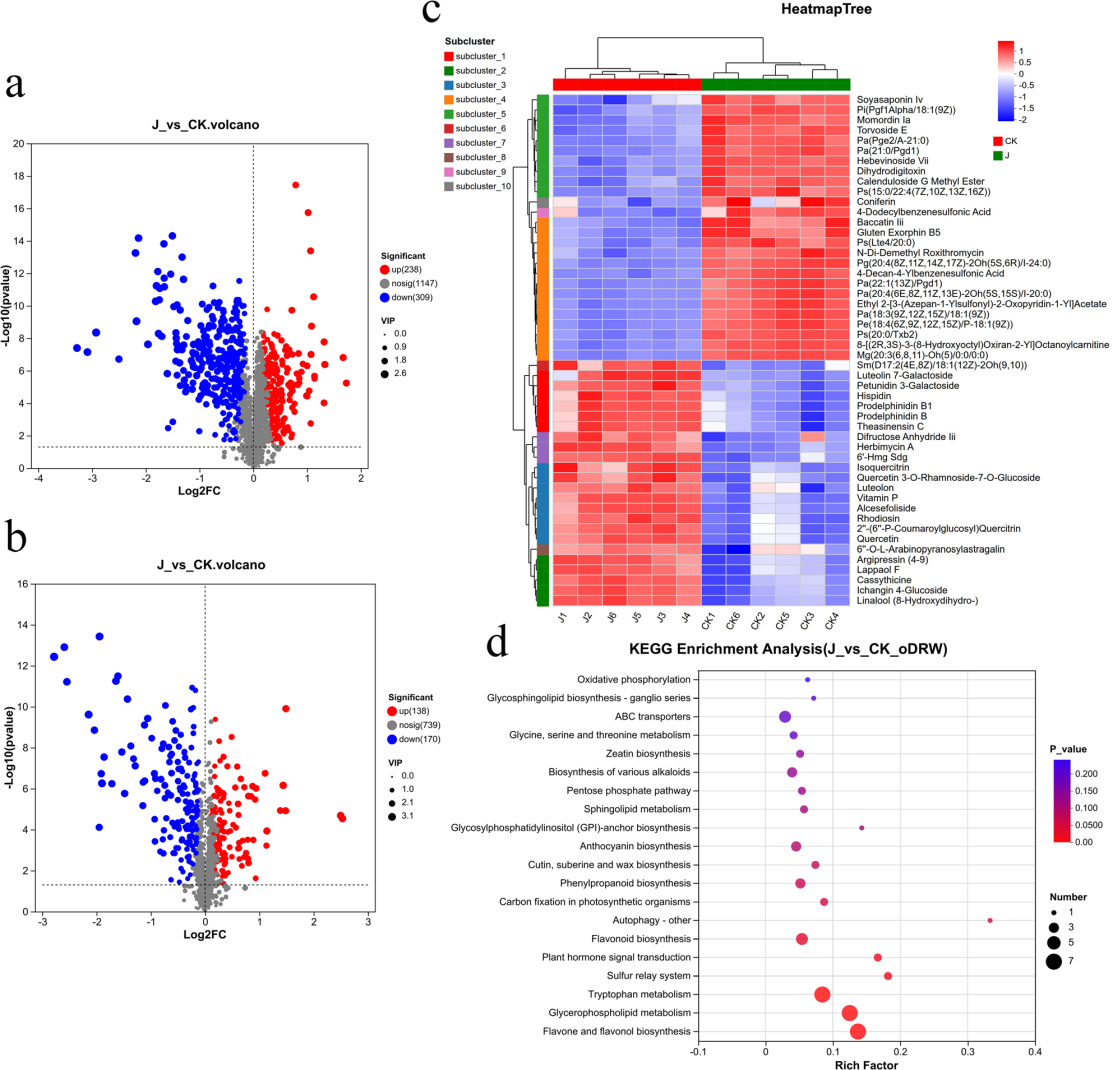
Analysis of differential metabolites and KEGG pathway enrichment in *G. biloba* leaves. **a**-**c**. **a** volcano plots differentially expressed metabolites in positive ion mode; (**b**) Volcano plots of differentially expressed metabolites in negative ion mode. Each point in the plots represents a metabolite, with red color being significantly up-regulated and blue color being significantly down-regulated; (**c**) Hierarchical clustering heatmap of the top 50 metabolites. Each column represents a sample and each row represents a metabolite, red represents up-regulation and blue represents down-regulation, with the darker the color the higher level of metabolite expression; and (**d**) KEGG metabolic pathway enrichment bubble chart. The chart uses different colors to represent the size of the *p*-value and different bubble sizes to represent the number of differentially enriched metabolites in the pathway

Comparative analysis identified 855 significantly altered metabolites in total. In positive ion mode, 547 metabolites were differential (238 upregulated, 309 downregulated), while 308 were altered in negative ion mode (138 upregulated, 170 downregulated) (Fig. 5a, b). Hierarchical clustering of the top 50 differentially abundant metabolites showed distinct expression patterns between groups (Fig. 5c), with notable upregulation of defense-related secondary metabolites including Luteolin 7-Galactoside, Theasinensin C, Hispidin, and Petunidin 3-Galactoside.

Pathway enrichment analysis based on the KEGG database revealed that the differential metabolites were significantly enriched in several key metabolic pathways (Fig. 5d). The three most significantly altered pathways (*p* < 0.01) were flavone and flavonol biosynthesis, glycerophospholipid metabolism, and tryptophan metabolism (Table 1). In the flavone and flavonol biosynthesis pathway, all identified flavonoids and phenolic compounds were upregulated except for Apigenin-7-(2-O-apiosylglucoside). Conversely, glycerophospholipid metabolites were generally downregulated. The tryptophan metabolism pathway showed predominant upregulation of intermediates and derivatives, including indole compounds and organic acids.

**Table 1:** Summary of metabolites in significantly enriched KEGG pathways. Metabolites are listed with their expression trends (up/down) and pathway *p*-values

KEGG pathway name	Metabolite	Tendency	*p*-value
Flavone and flavonol biosynthesis	Vitamin P	Up	0.00001385
	Quercetin 3-O-α-L-rhamnoside	Up	
	3-O-methyl quercetin	Up	
	Quercetin	Up	
	Isoquercitrin	Up	
	Apigenin-7-(2-O-apiosylglucoside)	Down	
	Kaempferol-3-O-sophoroside	Up	
Glycerophospholipid metabolism	Pg (20:4(5Z,8Z,11Z,14Z)/20:1(11Z))	Down	0.00002601
	Pe (20:3(8Z,11Z,14Z)/18:1(11Z))	Down	
	Ps (20:4(8Z,11Z,14Z,17Z)/14:1(9Z))	Down	
	Pa (18:3(6Z,9Z,12Z)/20:5(5Z,8Z,11Z,14Z,17Z))	Down	
	LPC (17:0/0:0)	Up	
	Pg (22:5(4Z,7Z,10Z,13Z,16Z)/22:6(4Z,7Z,10Z,13Z,16Z,19Z))	Down	
	Citicoline	Up	
Tryptophan metabolism	Indole-3-acetamide	Up	0.0003288
	L-5-Hydroxytryptophan	Up	
	Tryptophanol	Up	
	4-(2-aminophenyl)-2,4-dioxybutyric acid	Down	
	Indole-3-acetic acid (IAA)	Up	
	3-(3-Indolyl)-2-oxopropanoic acid	Up	
	Kynurenic acid	Down	

These metabolic shifts indicate that elicitor induction enhances *G. biloba* defense through coordinated reprogramming of secondary metabolism, promoting the accumulation of antimicrobial flavonoids and phenolic compounds while potentially reallocating resources from primary metabolic pathways toward defense compound synthesis.

## Discussion

The evolutionary arms race between plants and their fungal pathogens has driven the refinement of complex immune systems, with the perception of conserved microbial signatures, known as PAMPs, being a cornerstone of this innate defense [11, 27]. Our study elucidates that a cell wall-derived elicitor from the necrotrophic fungus *N. clavispora* can be effectively deployed to prime this innate immunity in the ancient gymnosperm *G. biloba*, conferring robust resistance against leaf blight. By integrating physiological phenotyping with untargeted metabolomics, we move beyond a descriptive account of efficacy to uncover the multi-layered defense strategy orchestrated by this elicitor. The central paradigm emerging from our data is that the elicitor does not function as a direct antimicrobial agent but acts as a sophisticated immunological trigger, establishing a primed state that potentiates the host’s cellular machinery for a faster, stronger, and more durable defense response, a phenomenon aligning with the established concept of defense priming [10, 28, 29].

The unequivocal absence of any direct inhibitory effect on *N. clavispora* spore germination and mycelial growth is a foundational observation, firmly redirecting the mechanism of action from a direct toxicological interaction to a host-mediated induction of resistance. This is a classic hallmark of Pattern-Triggered Immunity (PTI), where the recognition of PAMPs by plant surface-localized pattern recognition receptors (PRRs) initiates intracellular signaling cascades that mobilize defenses without directly impairing the pathogen [14]. The impressive efficacy of our elicitor, which outperformed several commercial resistance inducers and matched the protective level of the potent fungicide carbendazim, underscores two critical points: first, the particular molecular composition of this fungal cell wall extract represents a potent blend of PAMPs, and second, the immune system of *G. biloba*, despite its phylogenetic antiquity, possesses a highly responsive and effective PTI apparatus. This successful vaccination of plants using a homologous elicitor derived from the pathogen itself suggests a highly co-evolved recognition system, where the plant immune receptors are finely tuned to the most conserved and telling signatures of its adversary, enabling a highly targeted and efficient immune activation [29].

The detailed temporal analysis of defense physiology reveals the hallmarks of a primed system operating with enhanced efficiency. In elicitor-primed, challenge-inoculated plants (T-2), the defense enzymes (POD, PAL, PPO, CAT) did not merely achieve higher peak activities but exhibited distinct, often biphasic, kinetic profiles compared to the non-primed, pathogen-stressed plants (CK-2). The dramatic amplification of POD activity and the enhanced, biphasic nature of CAT activity indicate a superior capacity to manage the reactive oxygen species (ROS) burst. This oxidative burst is a critical defense signal but must be precisely spatially and temporally controlled to avoid catastrophic cellular damage; the elicitor-induced priming appears to fine-tune this process. The sustained and significantly elevated activity of phenylalanine ammonia-lyase (PAL) is of paramount importance, as it represents the entry point into the phenylpropanoid pathway, a major metabolic route for the production of defensive compounds [30]. Crucially, the fact that application of the elicitor alone (T-1), in the absence of any pathogen, was sufficient to elevate the baseline levels of these key defense enzymes provides direct evidence for the primed state. The plant’s defensive machinery is pre-assembled or poised for rapid deployment, accounting for the accelerated and amplified response observed upon subsequent pathogen challenge.

This concept of a pre-activated, alert state is powerfully reinforced by the dynamics of malondialdehyde (MDA), a reliable molecular scar of oxidative membrane damage. The rampant accumulation of MDA in the pathogen-only control (CK-2) signifies successful fungal colonization and a failure of the plant’s antioxidant systems to contain the ensuing oxidative stress. In striking contrast, the elicitor-primed plants (T-2) successfully constrained this damage, exhibiting a later, lower peak in MDA content followed by a clear decline, a pattern indicative of active containment, detoxification, and cellular repair mechanisms being deployed more effectively. The finding that the elicitor itself (T-1) did not provoke oxidative stress confirms that priming is a metabolically economical state of readiness, not a costly, full-scale defensive activation. This aligns perfectly with the life history strategy of a long-lived perennial tree like *G. biloba*, which must judiciously manage energy resources over its lifespan, avoiding unnecessary defense costs in the absence of real threat.

Our untargeted metabolomics approach allowed us to transcend the correlation of physiological markers and map the comprehensive metabolic change that constitutes the functional backbone of the induced resistance. The clear separation between treated and control groups in the multivariate models signifies a wholesale reconfiguration of the *G. biloba* leaf metabolome. This metabolic reorganization represents a biological defense response initiated by *G. biloba* to resist *N. clavispora* infection upon induction by the elicitor. To cope with various biotic stresses in their environment, plants have evolved numerous adaptive mechanisms to ensure survival [31, 32]. One such mechanism involves the production of specific chemicals in response to pathogen attack, including a range of defense-related compounds such as anthocyanins, tannins, flavonoids, and lignin [33]. Flavonoids, in particular, play a crucial role in protecting plants against pathogen infection [34]. The results of this study showed that after elicitor induction and infection by *N. clavispora*, flavonoid metabolites such as quercetin, isoquercetin, and 3-O-methylquercetin exhibited an increasing trend, thereby promoting the synthesis of phenolic compounds [35]. It is hypothesized that the fungal elicitor may induce *G. biloba* to release flavonoids, enhance the synthesis of secondary metabolites, and improve resistance to pathogens, thereby mitigating further damage to the plant. Their accumulation, mechanistically linked to the observed sustained induction of PAL, erects a potent chemical barrier that likely impedes pathogen establishment and mitigates oxidative damage.

Concurrently, the significant upregulation of the tryptophan metabolism pathway unveils another layer of defense modulation. This pathway is a nexus for the biosynthesis of crucial molecules, including the auxin indole-3-acetic acid (IAA) and other indolic compounds, which are increasingly recognized as key players in modulating defense signaling networks, often through complex cross-talk with salicylic acid and jasmonic acid pathways [36–38]. The enrichment of the “Plant hormone signal transduction” pathway in our data further supports the involvement of such complex phytohormonal crosstalk in orchestrating the primed state. The general downregulation observed in glycerophospholipid metabolism is equally telling. It may reflect a strategic metabolic trade-off, where carbon and energy resources are diverted away from the synthesis of membrane structural components and towards the more immediately critical biosynthesis of phenolic and flavonoid defense compounds—a classic reallocation from growth-related to defense-related metabolism under biotic stress.

Therefore, we propose a unified model for elicitor-induced resistance in *G. biloba*: Perception of the fungal cell wall PAMPs initiates a priming state, pre-conditioning the defense signaling network and the phenylpropanoid biosynthetic machinery. Upon subsequent pathogen attack, this primed status enables a rapid and potentiated oxidative burst that is simultaneously and effectively contained by a pre-enhanced antioxidant enzyme system, thereby minimizing collateral damage to host cells (as evidenced by constrained MDA accumulation). In parallel, the sustained activation of PAL channels metabolic flux into the massive and prolonged accumulation of lignin for structural reinforcement and a diverse arsenal of phenolic/flavonoid compounds for chemical defense. This entire process is fueled by a systemic reprogramming of primary and secondary metabolism, prioritizing defense output. This coordinated, multi-layered response—spanning enhanced oxidative signaling, physical fortification, and chemical antagonism—creates a hostile cellular environment that effectively restricts pathogen colonization and disease progression.

Our study employed a focused, systems-level approach combining detailed physiology and untargeted metabolomics to define the primed state in *G. biloba*. While transcriptional analysis of classic defense marker genes (e.g., *PR1*, *PR2*) would be complementary, our functional data showing sustained PAL enzyme activity and the consequent accumulation of phenylpropanoid pathway end-products provides direct biochemical validation of a key defense pathway. Furthermore, the significant and coordinated metabolomic shifts observed, such as the uniform upregulation of metabolites within the flavonoid biosynthesis pathway, offer robust, multi-faceted evidence for a wholesale defense reprogramming rather than an isolated response. This integrated perspective validates our elicitor as a potent priming agent and provides a functional map of the induced resistant state. The mechanistic insights and metabolic framework established here directly inform the logical next steps for this research, including the precise identification of the active elicitor component(s) and the characterization of the corresponding signaling machinery, as outlined in the conclusions.

## Conclusions

In conclusion, our integrated study provides a mechanistic blueprint for fungal elicitor-induced resistance in a relict gymnosperm species. We demonstrate that a cell wall elicitor from *N. clavispora* serves as a potent priming agent, enhancing *G. biloba* defense through a synergistic potentiation of its antioxidant system and an extensive reprogramming of its metabolic landscape, centrally involving the phenylpropanoid and flavonoid pathways. These findings significantly advance our understanding of immune perception and response in non-angiosperm plants. Looking forward, critical questions remain: the precise identity of the bioactive molecule(s) within the elicitor complex, the characterization of the corresponding PRRs in *G. biloba*, the interaction between elicitor and PRRs, the early immune response and its evaluation, as well as the applicability of the observed metabolic changes in enhancing *G. biloba* resistance. Furthermore, field trials are essential to validate the durability and efficacy of this strategy under natural conditions. This research not only enriches fundamental plant immunology but also lays a concrete foundation for developing sustainable, elicitor-based “plant vaccine” strategies for the protection of high-value forest and horticultural trees, offering a promising alternative to conventional chemical pesticides.

## Data Availability

The datasets used and/or analyzed during the current study are available from the corresponding author on reasonable request.

## Abbreviations

PTI: Pattern-triggered immunity
PAMPs: Pathogen-associated molecular patterns
PRRs: Pattern recognition receptors
ROS: Reactive oxygen species
MAPK: Mitogen-activated protein kinase
SAR: Systemic acquired resistance
PPO: Polyphenol oxidase
SOD: Superoxide dismutase
POD: Peroxidase
CAT: Catalase
PAL: Phenylalanine ammonia-lyase
MDA: Malondialdehyde
